# Low-cost urban heat environment sensing device with Android platform for digital twin

**DOI:** 10.1016/j.ohx.2024.e00598

**Published:** 2024-10-19

**Authors:** Yonghun Cho, Sanghyun Kim, Jeongseop Lee, Dongwon Ko, Haesung Lee, Yunju Baek, Myungho Lee

**Affiliations:** a School of Computer Science and Engineering, Pusan National University, Busandaehak-ro 63beon-gil, Geumjeong-gu, Busan 46241, Republic of Korea; b Department of Environmental Engineering, Pusan National University, Busandaehak-ro 63beon-gil, Geumjeong-gu, Busan 46241, Republic of Korea

**Keywords:** Urban heat monitoring, Mobile system, Citizen science

## Abstract

The proper monitoring of heat and meteorological variables is essential for the well-being of residents of metropolitan areas. It is challenging to configure spatial heat variations in complex urban environments, even though the temporal variation of urban heat flux has been measured at several designated monitoring stations. Neither the budget nor existing techniques for efficient urban heat monitoring are sufficient for a digital twin of the urban heat environment. As a result, we have developed a low-cost monitoring system that can be easily integrated into a portable pedestrian device, kickboard, or electric bike. With this system, citizens can collect information about urban heat, such as air temperature, surface temperature, relative humidity, barometric pressure, light intensity, and micro-geophysical features including topological aspects and mobile information (e.g., three-dimensional accelerations). Citizens can participate in daily scientific activities using these devices, which facilitate data acquisition and information exchange in urban digital twin environments.

## Graphical abstract

**Figure:**
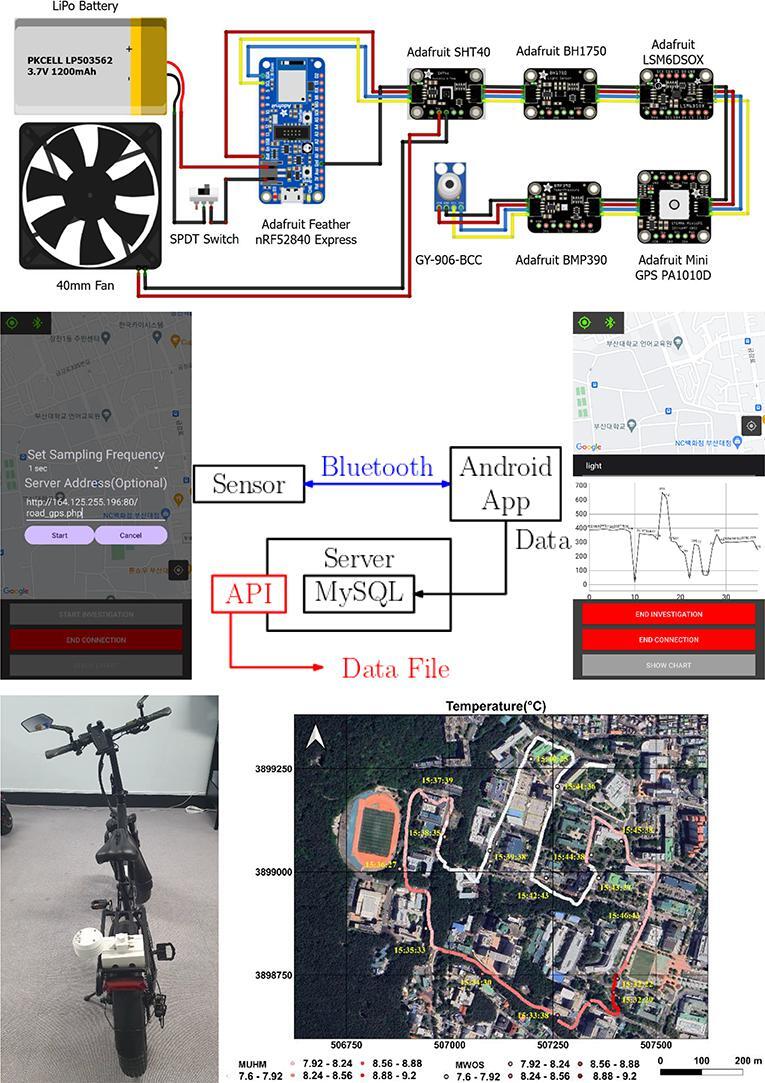

## 1 Specifications table

**Table:** 

Hardware name	Mobile urban heat monitoring system
Subject area	Environmental, planetary and agricultural sciences
Hardware type	• Measuring time series of air temperature, relative humidity, infrared surface temperature, illuminance, barometric pressure and gyro-sensor measurements (angles and accelerations in 3D) with GPS locations • Environmental engineering, electrical engineering, and computer science
Closest commercial analog	MeteoTracker X
Open-sources license	CC BY 4.0
Cost of hardware	128.71 USD
Source File Repository	https://doi.org/10.5281/zenodo.11086250

## 2 Hardware in context

Monitoring heat fluxes in urban environments is crucial to assessing and modeling urban heat islands [1], [2], [3], [4], [5], [6]. A mobile platform, which is proactive and effective, is essential for monitoring heat-related data on both spatial and temporal scales [1], [3]. A combination of mobile and fixed air temperature stations was explored in order to obtain a more accurate representation of urban heat flux spatially and temporally [1], [2], [5]. Considering the feasibility of data collection, public transportation (e.g., buses) has frequently been used for mobile heat monitoring. A representative path for the urban heat island can be used for repeated monitoring of heat flux [6], while additional data, such as surface temperature and barometric pressure, can also be useful for assessing the energy efficiency of buildings [7] as well as human temperature [8]. It has been reported that infrared sensors can be integrated with other sensors, such as humidity and illuminance, to estimate urban heat flux conditions in the field [9]. Furthermore, wearable air quality sensors have been tested for pedestrians as well as automobiles [3], [4].

Currently, mobile devices offer either basic monitoring parameters or are rather expensive and large for citizens to participate in citizen science. Current sensors are limited to a few commonly used meteorological variables (for example, temperature and humidity), and the path of mobile device is not specified without important micro information (for example, topography, velocity of the mobile system, surface temperature, and illuminance). Mobile systems can be scheduled and optimized to meet the demands of everyday life. A detailed analysis of mobile paths is crucial for advanced urban heat environment modeling, given the traffic in mega cities.

## 3 Hardware description

**Fig. 1:**
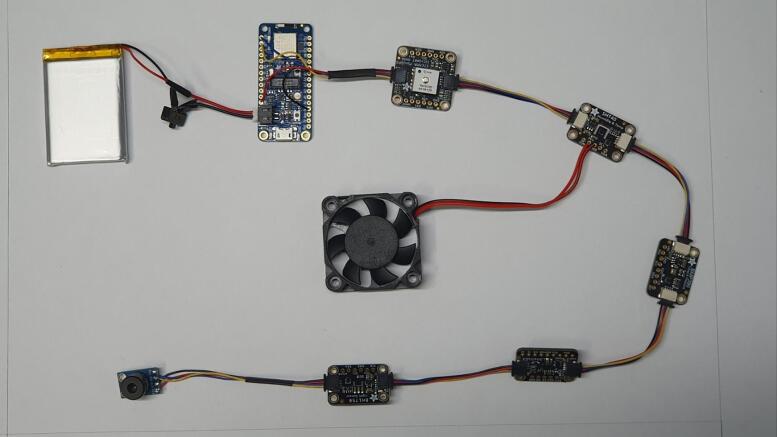
Composition of sensors, switch, fan and controller for mobile urban heat monitoring system.

Monitoring real-time heat environmental measurements has been implemented using a low-cost IoT prototype system (i.e., surface temperature by infrared sensor (℃), air temperature (℃), relative humidity (%), barometric pressure (hPa), illuminance (lx), accelerations (m/s^2^), and rotational motions (radian/s) in 3 dimensional axes with spatial coordinates from GPS in time series format). It can be equipped with a kickboard or an electric bike. In addition to carrying these items, pedestrians can also use simple accessories, such as smartphone sticks, to carry them. The collection of data and control of sensors can be carried out in two ways: through a serial Bluetooth terminal and through an APP developed for a smartphone. The apparatus consisted of a variety of devices (e.g., micro controllers, sensors, and a fan). An on/off switch was connected to a 3.7 Volt lithium polymer battery as a power supply. Fig. 1 shows how the mobile urban heat monitoring (MUHM) system is composed of electronic components.

### 3.1 Design files

The design files for the urban heat monitoring device are listed in Table 1, which serves as a repository of design files. A 3D printing design can be reproduced by using the files in Table 1. This repository also contained Android-based software for data transfer and communication, as well as firmware code for the controller.

## 4 Design files summary

**Table 1:** Urban heat monitoring design files.

Design file name	File type	Open source license	Location of file
Electric Schematic	JPG	CC BY 4.0	https://doi.org/10.5281/zenodo.11086250
Bluetooth Schematic	SVG	CC BY 4.0	https://doi.org/10.5281/zenodo.11086250
Breadboard	JPG	CC BY 4.0	https://doi.org/10.5281/zenodo.11086250
Firmware code	PY (Circuit Python)	CC BY 4.0	https://doi.org/10.5281/zenodo.11086250
Print3D (printing design)	ZIP(3MF)	CC BY 4.0	https://doi.org/10.5281/zenodo.11086250
Andriod software	ZIP(JAVA)	CC BY 4.0	https://doi.org/10.5281/zenodo.11086250
Data (case study database)	Serial.txt	CC BY 4.0	https://doi.org/10.5281/zenodo.11086250

## 5 Bill of materials summary

The bills of materials for the urban heat monitoring system are presented in Table 2 and includes controllers, sensors, and other devices. Table 3 summarizes accuracy of measurement parameters for sensors listed in Table 2. Most of these can be ordered from the corresponding web site.

**Table 2:** Urban heat sensor bill of materials.

Designator	Component	Number	Cost	Source of materials
Adafruit Feather nRF52840 Express	The Bluefruit feature with USB and CircuitPython support	1	24.95 $	https://www.adafruit.com/product/4062
GY-906-BCC	Melexis MLX90614 IR infrared temperature sensor	1	15.96 $	bit.ly/GY-906-BCC
Adafruit SHT40	Sensirion temperature/humidity sensor	1	5.95 $	https://www.adafruit.com/product/4885
Adafruit BH1750	BH1750 16-bit Ambient light sensor	1	4.5 $	https://www.adafruit.com/product/4681
AdafruitBMP390	BMP390 barometric pressure and altimeter sensor	1	10.95 $	https://www.adafruit.com/product/4816
Adafruit LSM6DSOX	6-Dof accelerometer and gyroscope sensor	1	11.95 $	https://www.adafruit.com/product/4438
Adafruit mini GPS PA1010D	MTK3333 chipset	1	29.95 $	https://www.adafruit.com/product/4415
Lithium Ion Polymer Battery	3.7v, 1200 mAh	1	9.95 $	https://www.adafruit.com/product/258
SPDT Slide Switch	Single pole double throw switch	1	0.95 $	https://www.adafruit.com/product/805
CFM-4010 V-070–273	40 mm 5 V DC Fans	1	5.69 $	bit.ly/CFM-4010V-070-273
STEMMA QT/Qwiic Cable	QT to QT cable	4	0.95 $	https://www.adafruit.com/product/4399
STEMMA QT/Qwiic Cable	QT to Female sockets	2	0.95 $	https://www.adafruit.com/product/4397

**Table 3:** Accuracy and function for sensors.

Sensor	Producer	Function	Accuracy
SHT40	Adafruit	Temperature	±0.2 °C
		Relative Humidity	±1.8 %
BMP390	Adafruit	Barometric Pressure	± 3 Pascal
		Altitude	± 0.25 m
MLX90614 IR	Melexis	Objective Temperature	±0.5℃
BH1750	Adafruit	Illuminance	lx variation ± 20 %
LSM6DSOX	Adafruit	Acceleration	±2/±4/±8/±16 g at 1.6 Hz to 6.7 KHz update rate
		Angular velocity	±125/±250/±500/±1000/±2000 dps at 12.5 Hz to 6.7 KHz update rate

## 6 Build instructions

**Fig. 2:**
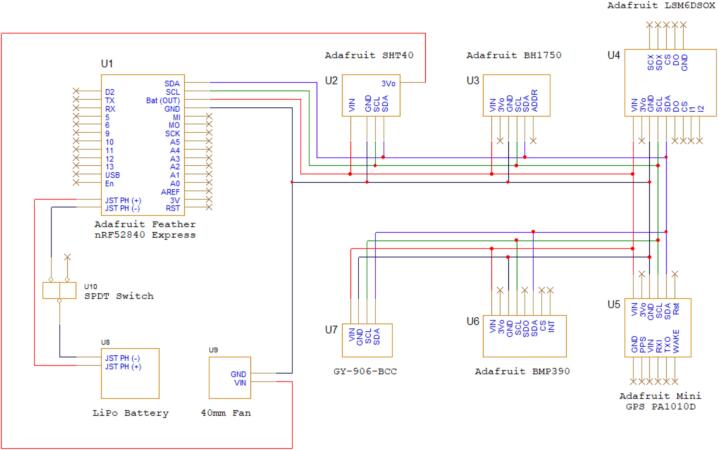
Electronic schematics showing wire connections between sensors and micro controller.

The Adafruit Feature nRF52840 Express board was used as the microprocessor of the system, which communicated with a smartphone via Bluetooth low-energy or native USB. GPS and time information can be obtained from a smartphone or the Adafruit mini GPS PA1010D. In addition to a USB type C connection to a smartphone battery, the system can also be powered by a lithium battery. The electronic schematics in Fig. 2 illustrate the connections between sensors and controllers.

**Fig. 3:**
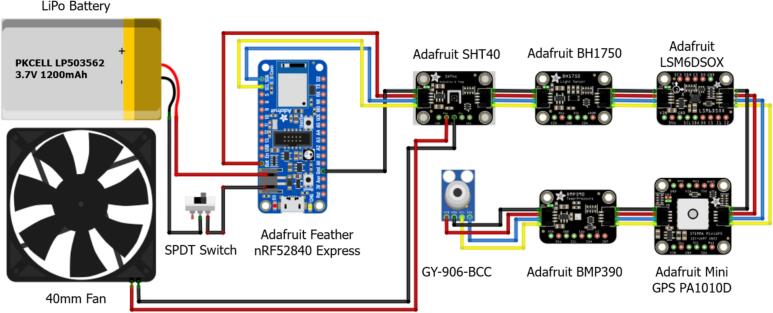
Schematics for sensor and controller connection lines for voltage in(red), ground(black), SDA(blue) and SCL(yellow) powered by lithium battery. (For interpretation of the references to color in this figure legend, the reader is referred to the web version of this article.)

A schematic of the breadboard for the circuit test can be found in Fig. 3, which shows the connections for power, ground, serial data (SDA) and serial clock (SCL). With a lithium battery as the power source, serial numbers of sensors, controllers, fans, and switches were presented with wire connections.

### 6.1 Fabrication of 3D printing layers for mobile urban heat monitoring (MUHM) system

**Fig. 4:**
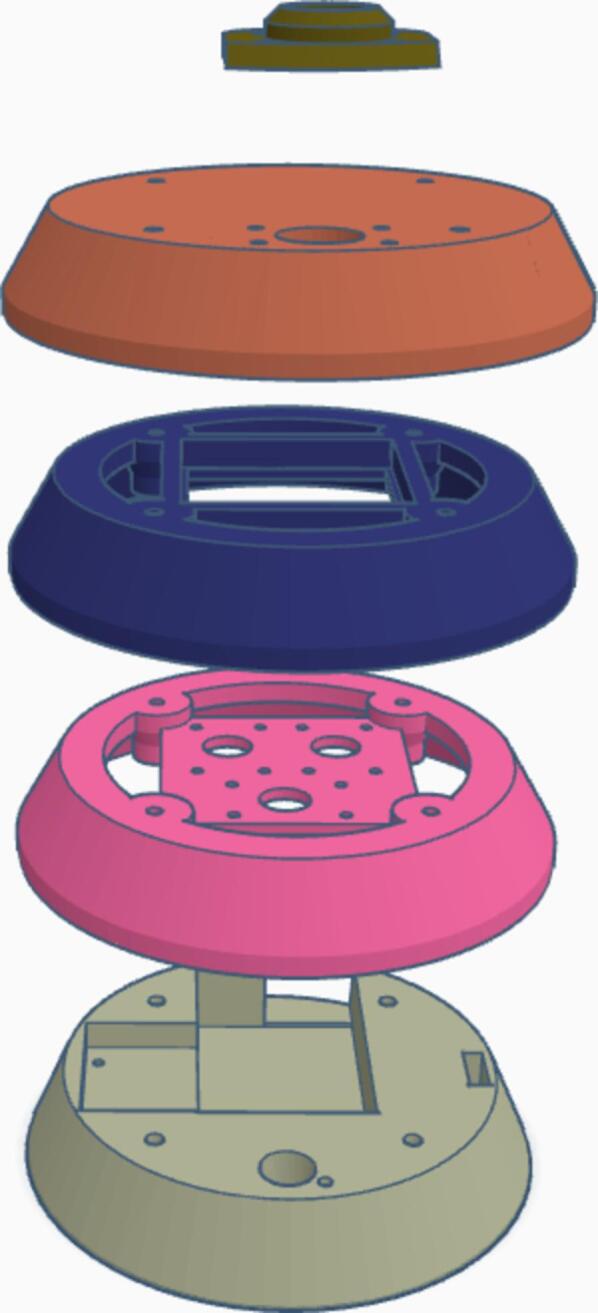
The assembly of 3D printed layers for Mobile Urban Heat Monitoring (MUHM) system.

**Fig. 5:**
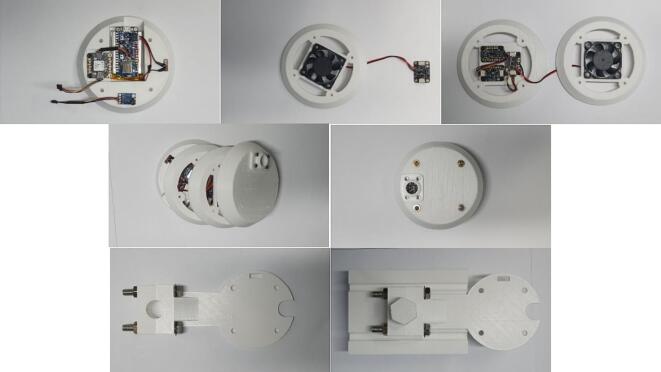
Assembling process for MUHM and attachment components for mobile platform.

Fig. 4 illustrates a four-layer 3D printing structure. For fabrication of Mobile Urban Heat Monitoring (MUHM) system, sensors and controllers shown in Fig. 2 were used. A controller, switch, lithium battery, PA1010D, and GY-906 are components of the 1st layer. By using two M2.5*8 bolts, PA1010D can be placed in the side space of the first layer. The GY-906 is fitted into a circle hole with the aid of a M3*8 bolt. A lithium battery and switch can be attached to the bottom of the first layer using a glue gun, and the controller can be stacked on top. The controller and PA1010D are connected with a STEMMA QT cable. The 2nd layer contains sensors of SHT40, BMP390, and LSM6DSOX. In the third layer, a square frame is fitted with a cooling fan, which is connected to the 3Vo and GND pins on the SHT40. Two M2.5*8 bolts are used to fix SHT40, BMP390 and LSM6DSOX, respectively. Sensors are connected using STEMMA QT cables. BH1750 and small cap (Fig. 4) are place into the circle hole in 4th layer using four M2.5*15 bolt and two 2.5 nuts. In order to mount four layers, four M3 bolts of 55 mm are used, and on the bottom layer, M3 nuts can be used. An assembly process for MUHM and component pictures for the attachment on the back of an electric bike are shown in Fig. 5.

**Fig. 6:**
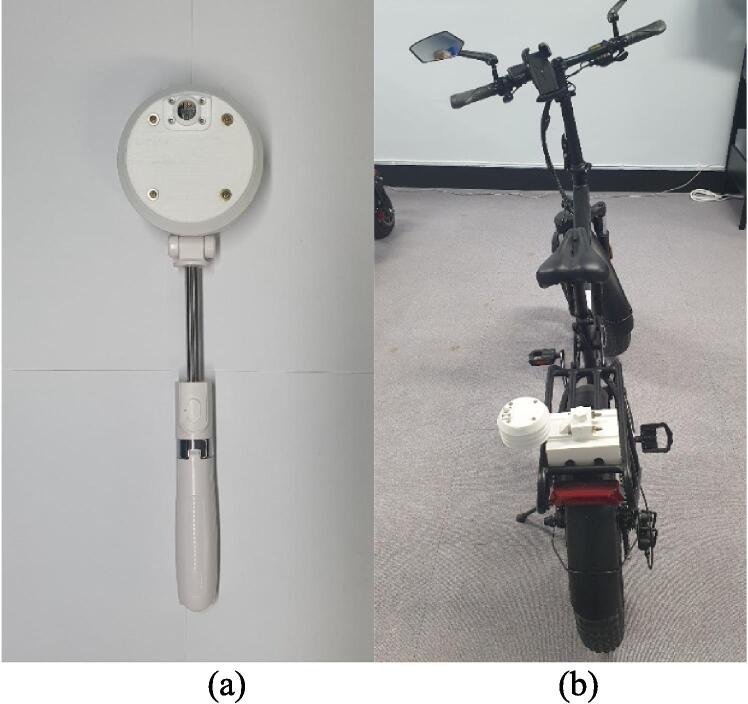
MUHM for pedestrian (a) and for electric bicycle (b).

The Fig. 6 shows MUHM systems for pedestrians (a) and electric bicycles (b). MUHM can be carried by hand or attached to a variety of mobile platforms.

### 6.2 The system for mobile urban heat monitoring

**Fig. 7:**
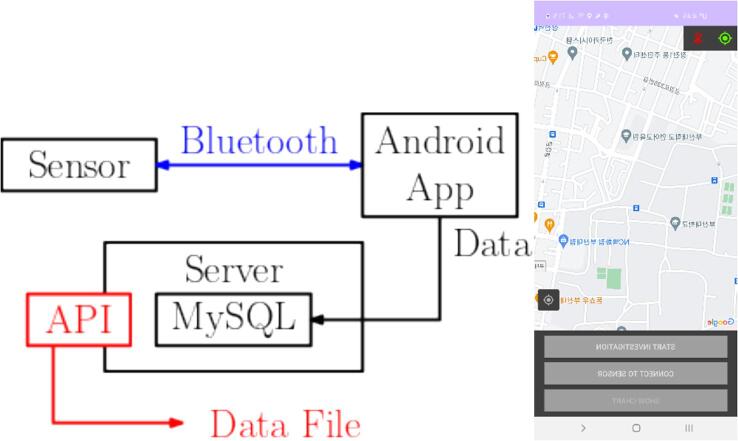
A schematic of API based centralization system and Android-based data-acquisition platform.

The measurement device was connected to each Android-based smartphone via Bluetooth, and the acquired data can be exchanged with other Android-based devices. All collected data is stored in an open API-based central system that is able to manipulate data collection and transfer. On each Android platform, the data are stored as time series with a reference time and location. An API-based centralized system and Android-based data acquisition platform are shown in Fig. 7.

## 7 Operation instructions

**Fig. 8:**
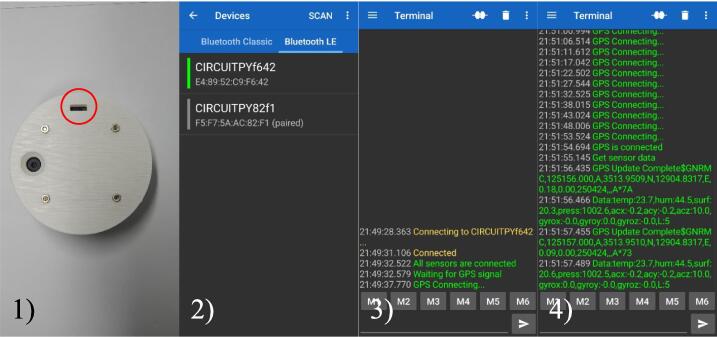
Procedure for MUHM activation 1), bluetooth selection 2), GPS connection 3) and data acquisition 4) using a serial Bluetooth terminal.

As the power of the system is activated, a low-energy Bluetooth connection can be authorized through an Android application. After a short waiting for the activation of GPS information, data from urban heat sensors can be monitored and recorded into the smartphone. Before staring the urban monitoring campaign, a checking procedure is essential to record data in the smartphone. The data acquisition procedure using serial Bluetooth terminal (Fig. 8) can be summarized in the following four steps:

1) Switch on and activate power of MUHM system

2) Connect using bluetooth between MUHM system and smartphone through Android APP.

3) Check the GPS and acquired data from the APP.

4) Perform field data monitoring and save measurements into the text format file.

**Fig. 9:**
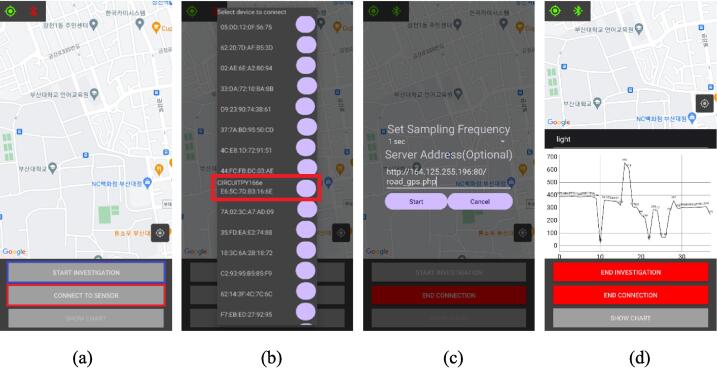
Sequential interfaces of developed APP for web server (a), device selection (b) sampling frequency (c) and data presentation (d).

An APP developed for the monitoring operation can also be used. Fig. 9 shows sequential interfaces for an APP. An active GPS enabled smartphone and a connection to the network (internet) are the first requirements. To connect to the sensor, click the red “CONNECT TO SENSOR” button in Fig. 9(a). Upon clicking, the device displays a list of connectable BLE devices in close proximity as shown in Fig. 9(b). You then need to select a device and connect the APP to the board. To collect data, press the blue “START INVESTIGATION” button in Fig. 9(a) after connecting. Fig. 9(d) shows that the two buttons turn red when the sensor is connected. After pressing the investigation button, the APP displays an option for collecting data (see Fig. 9(c)). There are two types of options: frequency of reading sensor data and web server address (DB) to send values.

In the absence of a web server, the address should be blank. During the investigation, data is transmitted to a web server and stored in the internal memory of the smartphone as a CSV file. Additionally, by pressing the “SHOW CHART” button while collecting data, the user can see the data shown as Fig. 9(d) in real-time. Once the data collection has been completed, click “END INVESTIGATION”. Use the “END CONNECTION” button to disconnect from the board.

## 8 Validation and characterization

### 8.1 Study area

**Fig. 10:**
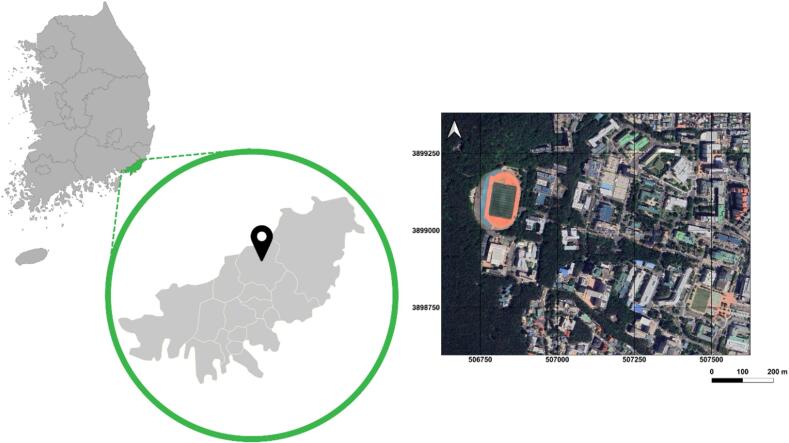
The location of study area (South Korea, Busan, and Pusan National University) and the study area with Universal Transverse Mercator (UTM) coordinate system.

The urban heat mobile monitoring system was tested as part of an urban safety Metaverse project. The study area was the main campus of Pusan National University in Busan, South Korea, situated on the mountainside of Mount Geumjeong. Fig. 10 shows the geographic location of the study area and the path of the field campaign. Data were recorded using an Android APP, which was implemented with the developed system.

**Fig. 11:**
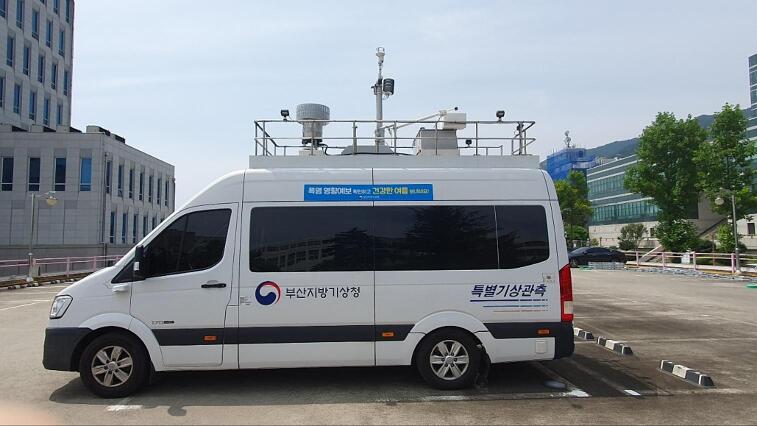
Korea Meteorological Administration’s mobile weather observation system (MWOS).

To compare the performance of the developed device, we also hired Korea Meteorological Administration’s mobile weather observation system (MWOS) (Fig. 11). There are several sensors on the roof of the vehicle that measure temperature, relative humidity, precipitation, pressure, wind direction, and wind speed. Mobile observation system is equipped with WL-T100 temperature sensor that measures temperatures from −70 °C to 100 °C with 0.3 °C accuracy. The WL-TH100 humidity sensor measures relative humidity (RH) from 0 to 100 % with an accuracy of 2 % (0–99 %) and a resolution of 0.1 %. The WLP-600-L pressure sensor has a working range of 300 to 1200 hPa and an accuracy of 0.2 %. Temperature, humidity, and pressure sensors receive electrical signals every 10 s, convert those signals into digital values, and average six sets of data collected every 10 s to produce minute data.

### 8.2 Data acquisition and analysis

**Fig. 12:**
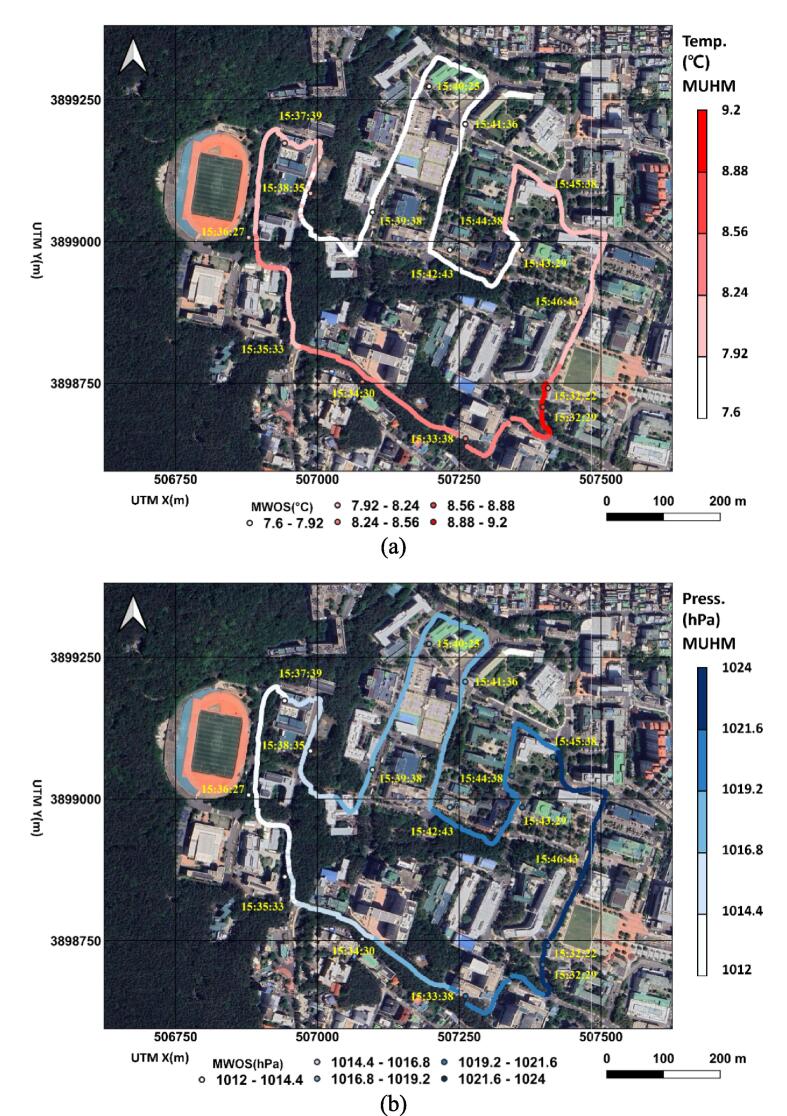

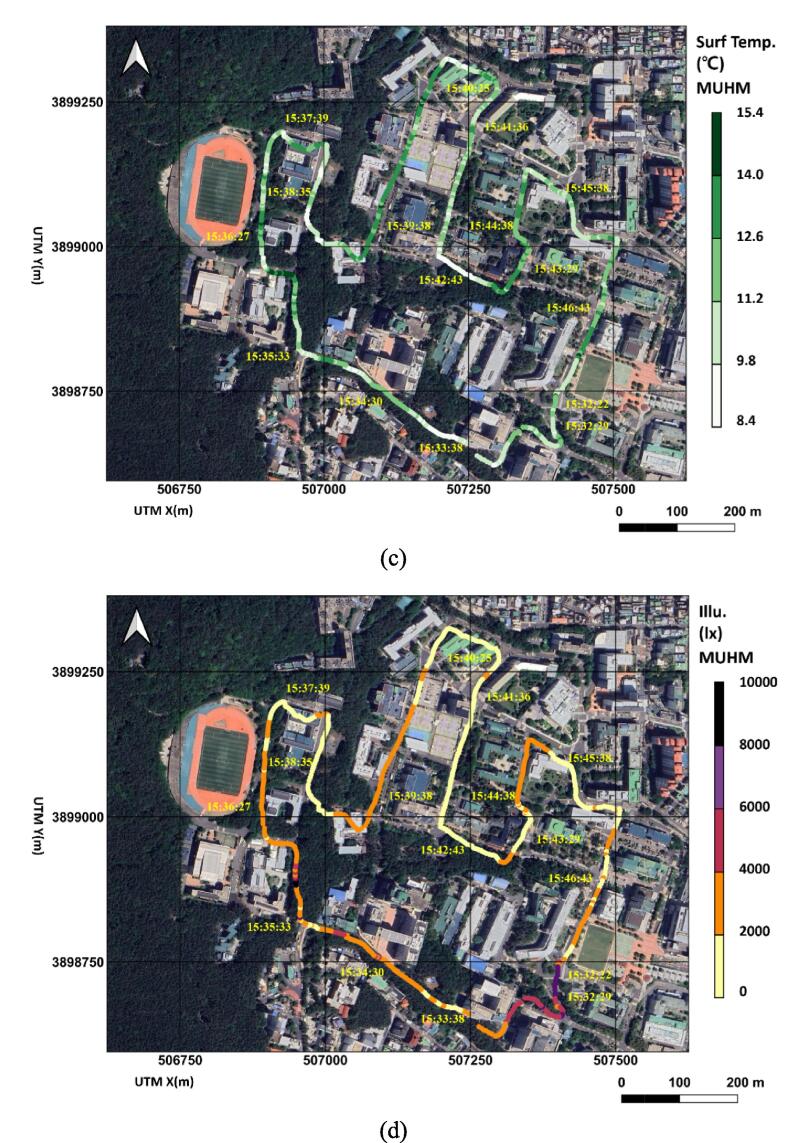
The urban weather monitoring results in terms of spatial distribution and temporal notations of air temperatures along route with asphalt pavement (℃) by MUHM and MWOS (a), barometric pressures (hPa) by MUHM and MWOS (b), surface temperatures (℃) by MUHM along route with asphalt pavement (c) and illuminances (lx) by MUHM (d).

The monitoring system transferred sets of real-time data for GPS location, time, air and surface temperatures, humidity, barometric pressure, illuminance (lx), and gyrosensor information (e.g., x, y, z accelerations in m/s^2^ and angular velocities in radian/s in three axes). In order to ensure proper data recording in smartphone storage, be sure that the monitoring device is connected to the smartphone APP. A field monitoring campaign was conducted on the path on 2nd Feb. 2024 for approximately 12 min (see Fig. 12). The sampling frequency of MUHM was 1 Hz, which can be changed depending on the route topography (e.g., degree of slope variation) and heterogeneity in environmental conditions (e.g., canopy, building, and road materials). Mobile routes have slopes ranging from 0 to 15 degrees. Shaded areas along the route due to canopy and buildings are divided into 46.8, 50.8, and 2.4 % of darker, intermediate and brighter parts, respectively. MWOS collected data in sampling frequency 0.0167 Hz. Considering the elevation difference of air temperature sensors between MUHM and MWOS, temperature measurements from MUHM were corrected to those from MWOS matching arithmetic means.

Fig. 12(a) shows the spatial and temporal notations of air temperature MUHM and MWOS. The data were collected and then corresponding images were generated with color scale later. Markers of pink colored circles and pink colored circles with black rings indicate MUHM and MWOS, respectively. Due to differences in sampling frequency, MUHM showed denser and continuous air temperature data, while MWOS showed discrete data. MUHM and MWOS had similar spatial distributions of air temperature, as shown in Fig. 12(a). The pressure distributions were shown in Fig. 12(b), which showed similar distributions between MUHM and MWOS. In Fig. 12(c) and (d), surface temperature and illuminance are presented spatially with temporal notation. A significant difference between the air temperature distribution and surface temperature amplitude range can be seen in Fig. 12(a) and (c). Due to substantial variations in canopy density, road surface slope, and surface aspect, which determine radiant energy, the variation rate of surface temperature was higher than that of air temperature.

We compared the air temperatures measured by MUHM and MWOS and evaluated the statistical difference between them (Table 4). As presented Table 4, the minimum and maximum differences in temperature and barometric pressure were less or more than the error bounds. Also, the RMSEs between MUHM and MWOS were lower than the accuracy ranges of each sensor.

**Table 4:** Minimum and maximum differences and root mean square error (RMSE) between MUHM and MWOS for air temperature and barometric pressure.

Parameter	Unit	Minimum difference	Maximum difference	RMSE
air temperature	℃	−0.2687	0.1636	0.1383
barometric pressure	hPa	−2.80	3.15	1.75

**Fig. 13:**
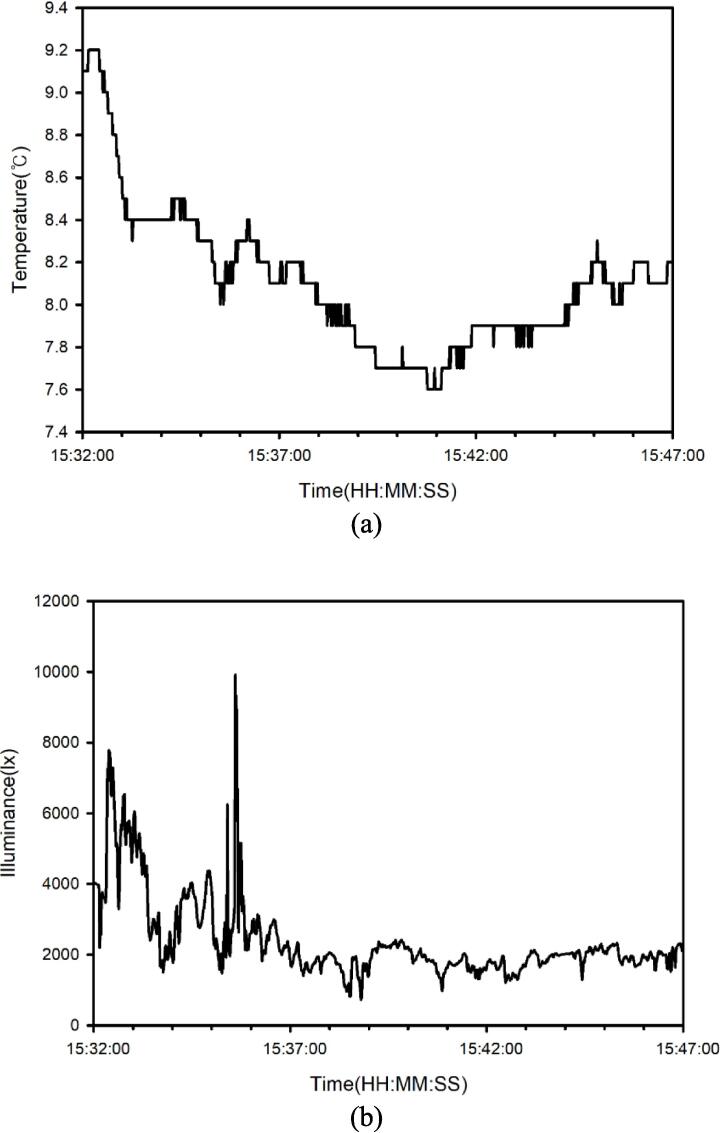

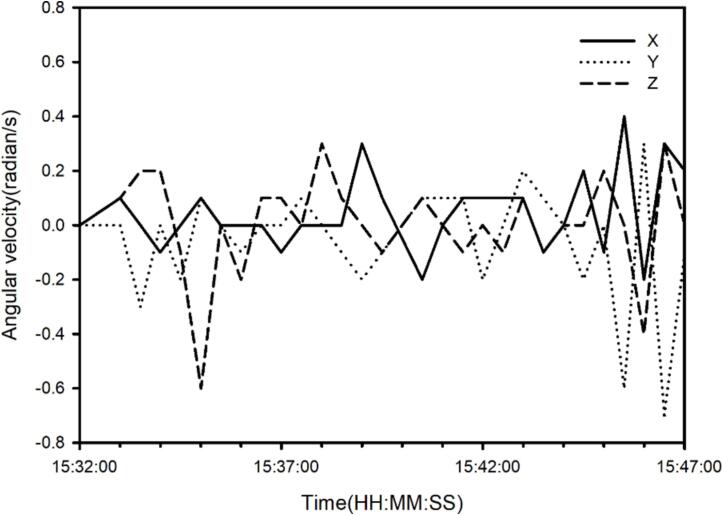
Time series of temperature in 1 Hz (a), illuminance in 1 Hz (b) and angular velocities (X, Y and Z axes) in 30 s interval (c) recorded from MUHM system.

Fig. 13(a) and (b) present time series of temperature and illuminance in 1 Hz. According to the Fig. 13(c), the surface slope varied substantially along the monitoring route as a function of the time series of 3D angular velocities recorded by the MUHM system. Fig. 13 (c) redraws angular velocity slope in 30 s intervals based on the data acquisition rate of 1 Hz. Slope variation data indicate tilt in road surface, which can be useful in evaluating meteorological processes (e.g., interaction between surface and solar radiation).

### 8.3 Power consumption

A lithium-ion battery powers the proposed system. Adafruit’s lithium-ion batteries have connectors that directly connect to the nRF52840 module, enabling easy power supply. A similar specification alternate lithium-ion battery was used instead of a lithium-ion battery due to shipping restrictions. If the connectors are not compatible, then a replacement of connectors is necessary to directly connect the module. Experimental results showed that a 1100 mAh battery could power the system for 11 h and 30 min. Using a 1200 mAh lithium-ion battery sold by Adafruit, the estimated operating time is 12 h and 30 min. Considering the multiple sensors, including GPS, the embedded system has a relatively high power consumption in comparison to other embedded systems.

### 8.4 Discussion and future work

There are a number of advantages to the proposed device with regard to sensor manipulation and operation protocols. It is possible to modify the hardware and software to meet specific needs and field conditions (e.g., topography, vegetation, and heterogeneity along the route). I2C STEMMA-QT connectors allow sensors, and relays to be connected quickly to controllers. The device functions are programmed in Circuit Python (CircuitPython), which can be easily modified to suit the operator’s preferences. Compared with commercially available meteorological devices (such as MeteoTracker X) [10], the developed device provides additional sensors for surface temperature (infrared sensor) and mobile parameters (gyroscope sensor) to track and monitor surface environmental factors at a lower cost than its commercial counterpart. Several other environmental sensors can be included in the system at reasonable costs, such as sound, magnetic field, proximity sensor, and fine particle sensor. Its Bluetooth communication with the Android App is another strength of the developed device. Mobile applications can be further developed to be able to connect with other applications as well as monitoring devices. Multiple monitoring programs can be coordinated through wireless communication between applications.

In order to build the meteorological digital twin in urban environments, the field data should be collected in real time. The measured data must be transmitted wirelessly to the server via mobile phone. Under an open API environment, visualized environmental layers (by physical modeling or statistical analysis) can be made and delivered to the user. In the development of urban weather digital twins, the developed device can play an important role.

The main limitation of the developed device is its power supply. A lithium battery was used to ensure field operation reliability. In order to ensure reliable data collection, voltage is checked and proper power bank management is ensured by using the developed device.

An integrated urban environmental monitoring system for digital twins is a valuable alternative to urban variable monitoring programs. The system successfully collects spatiotemporal variations in temperature, humidity, pressure, illuminance, and path information, such as angles and accelerations, in three dimensions. With this system, several context datasets are modeled in civilian resolution, and a variety of monitoring platforms, including pedestrians, mobile devices (including kick boards and electric bicycles), and conventional vehicles, are used to monitor urban heat variation. The enhanced digital twin for urban environments will need to optimize monitoring routes and schedules, control sampling frequencies in real-time, and coordinate multiple platforms for future development.

This project includes different areas and experts, such as environmental, urban, and electronic engineering, software development, and GIS, which demands collective experience for its implementation. This project can be easily modified and extended to address other urban environmental problems, including noise and fine particles. The low-cost urban environmental monitoring system facilitates the transfer of initiatives to citizen science activities through the engagement and training of stakeholders.

### CRediT authorship contribution statement

**Yonghun Cho:** Visualization, Methodology, Data curation. **Sanghyun Kim:** Writing – review & editing, Writing – original draft, Resources, Project administration, Funding acquisition, Formal analysis, Conceptualization. **Jeongseop Lee:** Formal analysis, Investigation, Visualization. **Dongwon Ko:** Formal analysis, Data curation. **Haesung Lee:** Formal analysis, Data curation, Software, Visualization. **Yunju Baek:** Supervision, Resources, Investigation, Funding acquisition. **Myungho Lee:** Supervision, Resources.

## Declaration of competing interest

The authors declare that they have no known competing financial interests or personal relationships that could have appeared to influence the work reported in this paper.
